# Multi-Walled Carbon Nanotube (MWCNT)-Reinforced Polystyrene (PS) Composites: Preparation, Structural Analysis, and Mechanical and Thermal Properties

**DOI:** 10.3390/polym17141917

**Published:** 2025-07-11

**Authors:** Kadir Gündoğan, Damla Karaağaç

**Affiliations:** Department of Nanotechnology Engineering, Uşak University, Uşak 64200, Türkiye; 1943050001@ogr.usak.edu.tr

**Keywords:** composites, carbon nanotube, plastic injection, thermal properties, mechanical properties

## Abstract

Polystyrene (PS), a thermoplastic polymer, is used in many applications due to its mechanical performance, good chemical inertness, and excellent processability. However, it is doped with different nanomaterials for reasons such as improving its electrical conductivity and mechanical properties. In this study, carbon nanotube (CNT)-added PS composites were produced with the aim of combining the properties of CNTs, such as their low weight and high tensile strength and Young’s modulus, with the versatility, processability, and mechanical properties of PS. In this study, multi-walled carbon nanotube (MWCNT)-reinforced polystyrene (PS) composites with different percentage ratios (0.1, 0.2, and 0.3 wt%) were prepared by a plastic injection molding method. The mechanical, microstructural, and thermal properties of the fabricated PS/MWCNT composites were characterized by Scanning Electron Microscopy (SEM), Fourier Transform Infrared (FTIR) Spectroscopy, Atomic Force Microscopy (AFM) and Thermogravimetric Analysis (TGA) techniques. AFM analyses were carried out to investigate the surface properties of MWCNT-reinforced composite materials by evaluating the root mean square (RMS) values. These analyses show that the RMS value for MWCNT-reinforced composite materials decreases as the weight percentage of MWCNTs increases. The TGA results show that there is no change in the degradation temperature of the 0.1%- and 0.2%-doped MWCNT composites compared to pure polystyrene, but the degradation of the 0.3%-doped MWCNT composite is almost complete at a temperature of 539 °C. Among the PS/MWCNT composites, the 0.3%-doped MWCNT composite exhibits more thermal stability than pure PS and other composites. Similarly, the values of the percentage elongation and tensile strength of 0.3% MWCNT-doped composites was obtained as 1.91% and 12.174% mm^2^, respectively. These values are higher than the values of 0.1% and 0.2% MWCNT-doped composite materials. In conclusion, the mechanical and thermal properties of MWCNT-reinforced PS polymers provide promising results for researchers working in this field.

## 1. Introduction

Polymer materials are widely used in many engineering applications, such as in the aerospace industry [1], electronics and energy storage [2], automotive industry [3], and biomedical applications [4], due to their superior properties, such as mechanical strength, processability, and light weight [5,6]. Among them, thermoplastic polymers such as polystyrene (PS) play an important role in various fields, such as composites, packaging, energy storage, construction, and machine elements [3]. In some applications, however, pure polystyrene’s mechanical and thermal properties are insufficient [4]. Therefore, polystyrene needs to be reinforced with various reinforcement materials to improve its performance. In recent years, the mechanical and thermal properties of polymer composites have been significantly improved through the use of nanomaterials such as MWCNTs [5,6].

MWCNTs can be incorporated into polymer matrices to improve the overall performance of composites, with properties such as a high mechanical strength and low density [7,8]. The distribution and interactions of MWCNTs in the polystyrene matrix are important factors affecting the performance of composites.

Specifically, the interaction of MWCNTs with polystyrene increases the glass transition temperature by limiting the mobility of the polymer chains, which has a positive effect on the thermal stability of the composites [9,10]. In the literature, studies on the mechanical properties of polystyrene composites reinforced with MWCNTs have shown that MWCNTs increase the durability and the thermal stability of such composites. In particular, the integration of MWCNTs into the polystyrene matrix increases the tensile strength and elongation percentage of the composites, making them more suitable for engineering applications [11,12].

However, the uniform distribution of MWCNTs in the polystyrene matrix is critical for improving the mechanical properties. A uniform distribution improves the overall durability of the composites by enhancing load transfer [13,14]. Moreover, the thermal conductivity of composites obtained by the addition of MWCNTs is also significantly increased. This provides a great advantage especially for heat management applications. The thermal conductivity properties of MWCNT-reinforced polystyrene composites show a significant improvement over conventional polymer materials [15,16]. This property offers potential applications in cooling and heat dissipation of electronic components.

In addition, it should be emphasized that MWCNT-reinforced polystyrene composites have a significant potential in terms of environmental sustainability. Recycling and reuse of polymer wastes has become a critical requirement to tackle environmental issues today. The use of MWCNTs improves the mechanical and thermal properties of polymers, resulting in higher performance with less material. This offers significant economic and environmental advantages [17,18].

In this study, CNTs were reinforced into pure polystyrene at different percentages (0.1, 0.2, and 0.3 wt%) and PS/MWCNT composites were produced by plastic injection molding method. SEM analysis, FTIR, and AFM techniques were used for the microstructural characterization of these composite materials, while tensile tests and TGA analysis were performed to determine the mechanical and thermal properties, respectively. In the mechanical tests, it was observed that MWCNT reinforcements at different ratios had a positive effect on the mechanical properties of the composites. Furthermore, this study provides important contributions on the preparation and characterization of MWCNT-reinforced polystyrene composites. In particular, the detailing of the methods used for the homogeneous dispersion of nanofillers into the polystyrene matrix fills gaps in the existing literature and increases the knowledge in this field. It should also be emphasized that the results obtained provide a significant advance in understanding the thermal and mechanical properties of MWCNT-PS systems and point to potential new applications.

Although the reinforcement of polystyrene (PS) with multiwalled carbon nanotubes (MWCNTs) has been widely investigated, this study offers a different perspective by optimizing dispersion techniques and evaluating mechanical and thermal performance under controlled processing conditions. At the same time, injection molding parameters for enhanced MWCNT dispersion were studied and MWCNT loading thresholds were systematically evaluated. As a result, a potential composite material compatible with recent developments in aerospace and electronics applications and for high-performance engineering solutions was produced.

## 2. Materials and Methods

### 2.1. Materials

The crystallized polystyrene (CAS No: 100-42-5) used in this study was obtained from Izmir Dinamik Isı (Izmir, Turkey). Polystyrene is a thermoplastic polymer widely used in various engineering applications. The high processability and light weight of polystyrene make it an ideal material for many industrial applications. The molecular weight of the polystyrene used in this study was predicted as approximately between 98,000 and 105,000 g.mol^−1^. In addition, the MWCNTs (CAS No: 308068-56-6) used in this study were obtained from Nanography Nano Technology (Ankara, Turkey). MWCNTs are known for their properties, such as high mechanical strength and low density, and are used as a reinforcement material in polymer matrices. The purity of the MWCNTs used is 92% and their outer diameter ranges between 8–10 nm.

### 2.2. Plastic Injection Molding Method

In this study, commercially available crystalline polystyrene and multi-walled carbon nanotube nanoparticles were used as reinforcing elements. The nano-reinforcements were added to pure polystyrene with weight percentages of 0.1%, 0.2%, and 0.3% and the materials were produced by a plastic injection molding method. In the plastic injection molding process, specific values were used for some of the processing parameters, such as temperature settings, injection speed, mold temperature, mixing time, and mold dimensions.

The injection molding machine was set to a temperature between 200–220 °C to melt the polystyrene. This range was chosen based on the thermal properties of polystyrene (PS) and aligns with the processing parameters documented in prior studies on PS/MWCNT nanocomposites [19,20]. This temperature increases the fluidity of the polystyrene, enabling smooth pouring into the molds. The injection speed was set at 50–80 cm^3^/s to ensure complete mold filling without inducing thermal degradation, consistent with the practices reported by Mathur et al. [21]. To control the cooling process, the mold temperature was kept between 30 and 50 °C. This range promotes uniform solidification and reduces residual stresses during polymer crystallization [22]. During mixing, the pre-compounding process was conducted at 80 rpm for 10 min. This speed was chosen to promote the homogeneous dispersion of the MWCNTs, as described in other melt-mixing studies [23,24]. The mixed materials were then poured into pre-prepared molds measuring 50 × 130 mm. This size provides suitable geometry for mechanical testing according to ASTM standards.

**Figure 1:**
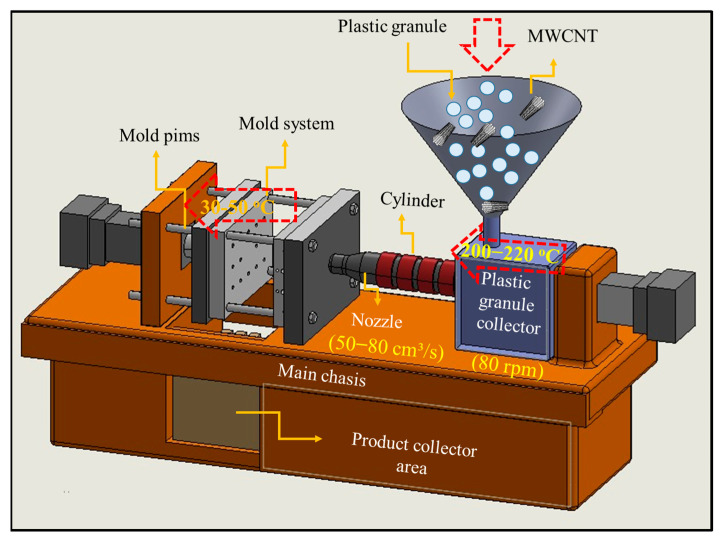
Schematic representation of the experimental process during the producing of the PS/MWCNT nanocomposites.

The produced composite materials were firstly investigated by microstructure characterization, i.e., SEM and AFM analyses, and their mechanical properties were investigated by hardness and tensile tests. The experimental process during the producing of the PS/MWCNT nanocomposites is presented schematically in Figure 1.

### 2.3. Characterization Techniques

The surface morphology of the material and the distribution of MWCNTs in the polystyrene matrix were investigated using a HITACHI SU5000 FE-SEM (Ankara, Turkey). In this context, the surface morphology of the PS/MWCNT composites was obtained by utilizing the AFM Workshop TT non-contact mode. The average roughness, root mean square, and *z*-height values of these composites were recorded in non-contact mode and 5 mm × 5 mm in scaling size. Additionally, a Hitachi STA7000 TGA (Usak, Turkey)was used to determine the thermal stability of the polystyrene and MWCNT composites by examining the mass loss of the material with increasing temperature. The TG (weight loss) curves of PS/MWCNT composites were measured from room temperature to 1000 °C. FTIR is a significant instrument for the observation of functional groups that are involved in the stabilization of materials. A Perkin Elmer Spectrum Two FT-IR Spectrophotometer (Usak, Turkey) was used to reveal clear peaks of PS, MWCNTs, and PS/MWCNT composites throughout the whole range of observation in spectra of 4000–450 cm^−1^.

## 3. Results and Discussion

### 3.1. SEM and AFM Results of Pure PS and PS/MWCNT Composites

**Figure 2:**
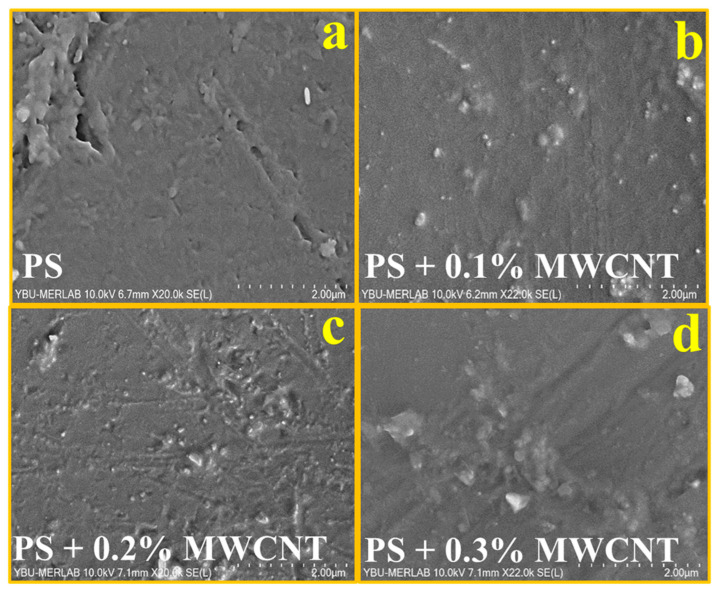
SEM images of pure PS (**a**), PS + 0.1% MWCNT composite (**b**), PS + 0.2% MWCNT composite (**c**), and PS + 0.3% MWCNT composite (**d**).

In order to obtain the surface images of the PS and PS/MWCNT composite materials, SEM analyses were performed with a Hitachi SU5000 (Ankara, Turkey) brand device. Figure 2a shows that the SEM image of pure polystyrene has a homogeneous distribution. The SEM images of PS/MWCNT composites in Figure 2 display that MWCNT nanoparticles are in harmony with the matrix material and have a uniform distribution. In general, the reinforcement materials were homogeneously distributed in the polymer matrix. The surface interaction between the polymer and reinforcement materials during the formation of the composite material had an effect on the homogeneous distribution of the materials.

**Figure 3:**
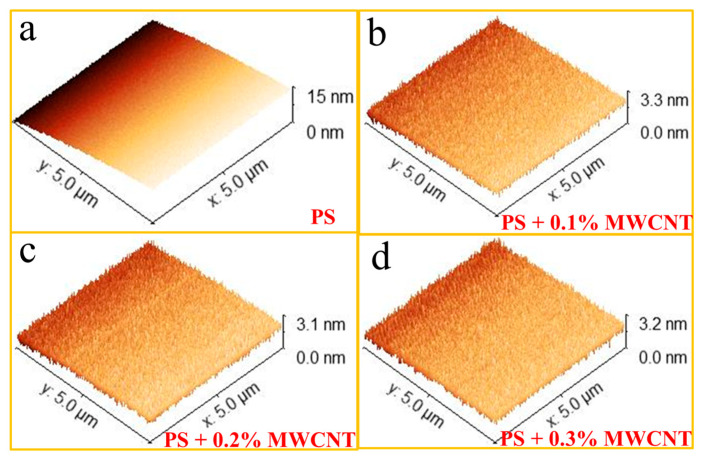
AFM images of pure PS (**a**), PS + 0.1% MWCNT composite (**b**), PS + 0.2% MWCNT composite (**c**), and PS + 0.3% MWCNT composite (**d**).

Morphological examinations of pure polystyrene and 0.1, 0.2, and 0.3% MWCNT-reinforced composite materials were carried out with the TT-2 AFM microscope and are given in Figure 3. Three-dimensional images of composite samples pressurized in discs are shown with an area of 5 µm × 5 µm. The values of Root Mean Square (RMS), Sq, and Mean Roughness, Sa, were obtained to investigate the surface properties of pure PS and the PS/MWCNT composites’ parameters.

The RMS (Sq) values of pure PS, PS + 0.1% MWCNTs, PS + 0.2% MWCNTs, and PS + 0.3% MWCNTs were observed as 3.775 nm, 0.457 nm, 0.446 nm, and 0.411 nm, respectively. The Mean Roughness (Sa) values of pure PS, PS + 0.1% MWCNTs, PS + 0.2% MWCNTs, and PS + 0.3% MWCNTs were observed as 3.252 nm, 0.369 nm, 0.364 nm, and 0.332 nm, respectively. AFM results showed that RMS values decreased with increasing reinforcement percentage in the reinforced composites compared to pure polystyrene. In other words, as both reinforcement ratios increase, the roughness of the material surface decreases significantly. In addition, the three-dimensional AFM images of the composite samples coincide with the SEM images we have previously obtained and show that the reinforcement materials are homogeneously distributed in the composite.

Additionally, the decrease in RMS roughness values as MWCNT content increases indicates enhanced surface homogeneity and improved nanotube dispersion within the polystyrene (PS) matrix. This reduction in surface irregularities minimizes stress concentration points, resulting in enhanced mechanical performance, particularly tensile strength and strain distribution. Further, a smoother, denser surface morphology may reduce phonon scattering and void formation, thereby improving thermal conductivity and stability. These results align with those of previous studies on well-dispersed nanocomposites [25].

### 3.2. FTIR Analyses of Pure PS and PS/MWCNT Composites

**Figure 4:**
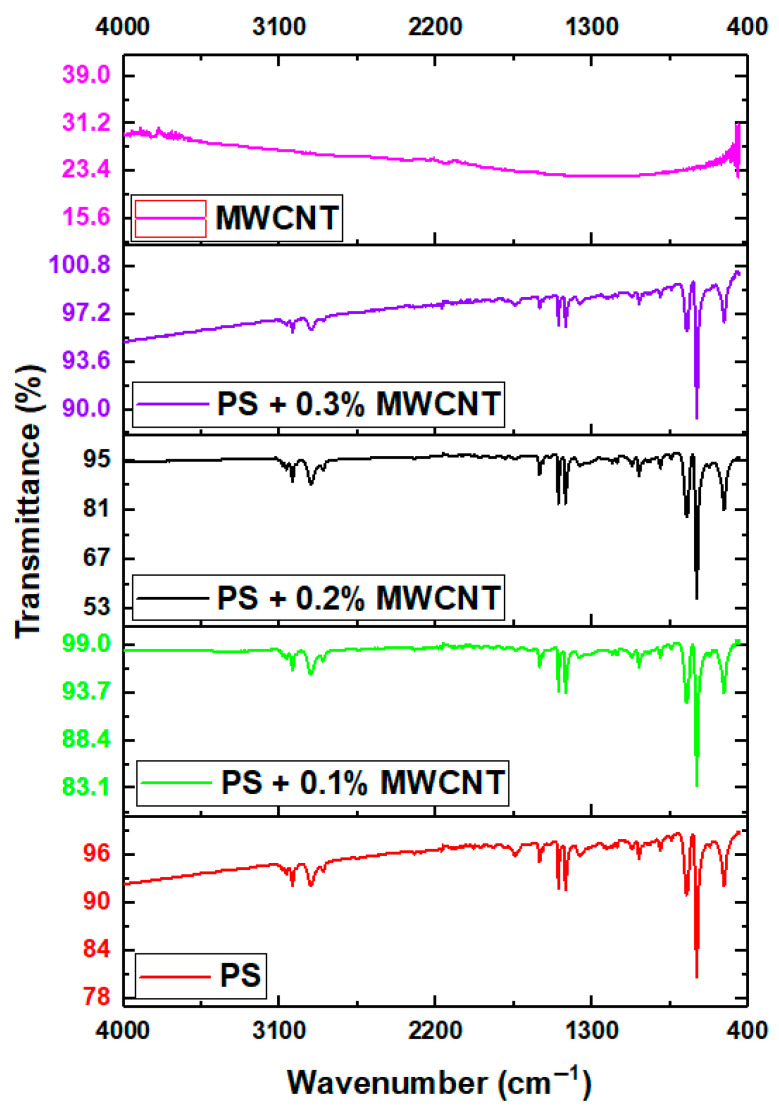
FTIR analyses of pure PS, MWCNTs, and PS/MWCNT composites.

FTIR analysis was performed on pure PS and PS/MWCNT composites reinforced with MWCNTs at different rates of 0.1, 0.2, and 0.3%. Figure 4 shows FTIR analyses of pure PS and PS/MWCNT composites. There is no significant change in the PS characteristic peaks in the FTIR spectrum of PS/MWCNT composites, except for slight shifts in some C-H bands. The peaks at 3026.21 cm^−1^, 2919.84 cm^−1^, and 2846.17 cm^−1^ have been assigned to a C-H aliphatic and aromatic bond stretching in accordance with the literature [26]. The strong absorption band that appeared at 1601.18 cm^−1^ has been attributed to a C=C bond, while the peak at 1493.16 cm^−1^ is arising from the C=C stretching of benzene-rings. For these peaks, it was found that the values were very similar to the PS-FTIR analysis peak values in the literature [27]. The peaks at 1451.89 cm^−1^, 1154.78 cm^−1^, and 1068.86 cm^−1^ have been assigned to CH_2_ + C=C bond stretching, C-O bond stretching, and C-O-C bond stretching, respectively, and are compatible with previous works [26]. Other peaks that appeared at 1028.17 cm^−1^, 753.13 cm^−1^, and 694.76 cm^−1^ have been attributed to C-H bond stretching.

### 3.3. TGA Analyses of Pure PS and PS/MWCNT Composites

**Figure 5:**
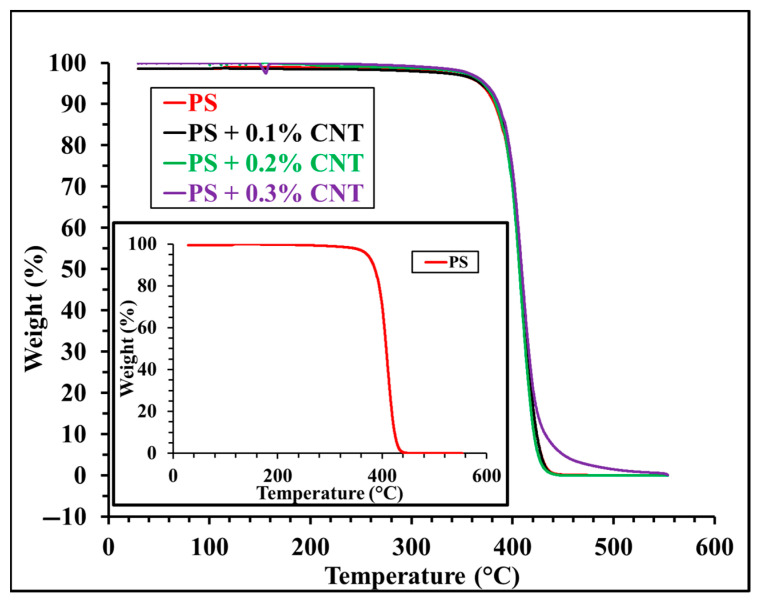
TG analyses of pure PS (inset) and PS/MWCNT composites.

Thermogravimetric analysis (TGA) measures the numerical value of the weight loss that occurs with temperature in the quantity of a sample. The mass change of a sample during water loss and degradation can be observed as a function of time and temperature. With increasing temperature, mass loss occurs as a result of the breaking of bonds in chemical and physical reactions. The graph of weight and weight percentage versus time is called a thermogram or thermal degradation curve. TGA was performed on pure PS and PS/MWCNT composites reinforced with MWCNTs at different rates of 0.1, 0.2, and 0.3%. Figure 5 shows the TG analyses of the pure PS and PS/MWCNT composites. The thermal decomposition of each sample was carried out over a programmed temperature range of 30–600 °C. Figure 5 shows that the degradation temperature of pure PS started at 298 °C and was completed at a temperature of 437 °C, which indicates a better thermal stability for pure PS. Similar observations are presented in previous studies where the maximum temperature of decomposition of PS is contained between 425 and 429 °C depending on the analysis conditions [28]. On the other hand, there was no change in the degradation temperature of 0.1%- and 0.2%-doped MWCNT composites compared to pure polystyrene. However, the decomposition of the 0.3%-doped MWCNT composite was almost complete at a temperature of 539 °C. The decomposition temperature of CNTs in TGA is between 500 and 600 °C [29]. Therefore, by increasing the CNT ratio to 0.3 wt%, the sample remained thermally stable up to higher temperatures. This shows that the addition of CNTs into PS increases thermal stability [30]. As a result, the thermal stability of the 0.3%-doped MWCNT composite is higher than pure PS, and MWCNT reinforcement had a positive effect on the decomposition temperature of PS polymer.

### 3.4. Tensile and Hardness Tests of Pure PS and PS/MWCNT Composites

**Figure 6:**
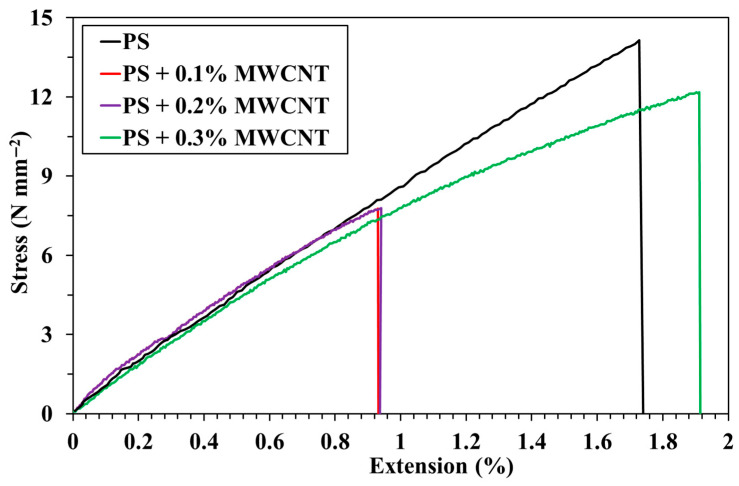
Tensile test results of pure PS and PS/MWCNT composites (average of five specimens per group, in accordance with ASTM D3039).

Tensile testing is a basic materials science test in which a specimen is subjected to an axial tensile force until it breaks. The results obtained from the test are used for material selection for any application, quality control, and to predict how the material will behave under other forces [31]. Tensile tests were performed on pure PS and PS/MWCNT composites reinforced with MWCNTs at different rates of 0.1, 0.2, and 0.3 wt%. Each sample group was tested five times and the average values were calculated in accordance with ASTM D3039. Figure 6 shows the results of tensile tests of pure PS and PS/MWCNT composites. These results were presented in terms of two important mechanical parameters, namely the percentage elongation value and tensile strength.

The values of the percentage elongation and tensile strength were obtained as 2.90% and 14.15 N/mm^2^ for the pure PS, respectively. The percentage elongation value of 0.93% was obtained in composites with 0.1% MWCNT additives, while the tensile strength was read as 7.75 N/mm^2^.

Figure 6 also shows the tensile test graph of the 0.2% MWCNT-reinforced composite. From this graph, it is observed that both the percentage elongation and tensile strength values of the 0.2% MWCNT-reinforced composite remain the same as the 0.1% MWCNT-reinforced composite. On the other hand, the values of the percentage elongation and tensile strength of 0.3% MWCNT-doped composites were obtained as 1.91% and 12.17 N/mm^2^. The superior tensile performance that was observed at 0.3 wt% MWCNTs is likely due to improved dispersion and stronger interfacial bonding between the nanotubes and the polymer matrix, which facilitates efficient stress transfer [32]. In contrast, the diminished or unchanged tensile behavior at lower loadings (0.1% and 0.2%) may be attributed to MWCNT agglomeration. This is because insufficient interfacial interaction hinders load transfer. This creates localized stress concentrations. These values are higher than those of 0.1% and 0.2% MWCNT-doped composite materials.

Based on the tensile test results, the percentage elongation and tensile strength values decreased in MWCNT-doped composites compared to pure polystyrene. In other words, since MWCNTs hardened the material, the flexibility of the material decreased.

Similar results were reported by Kaseem et al. [19], who investigated the effects of SWCNT content on the mechanical properties of PS/SWCNT composites. Similar results were reported in the study by Kaseem et al. [19], which investigated the effects of SWCNT content on the mechanical properties of PS/SWCNT composites. In their study, the flexural strength and strain-at-break values of PS composites reinforced with 0.1, 0.2, 0.3, and 1.0 wt% SWCNTs were investigated. PS reinforced with 0.1 wt% SWCNTs showed a significant decrease compared to pure PS. On the other hand, there was a gradual increase in flexural strength and tensile strain as the wt% SWCNT content increased.

A study presented by Rezakalla and Petrovna [20] investigated the tensile properties of recycled high- and low-density polyethylene. It was observed that the tensile strength decreased as the recycled content increased, but the MWCNT additive decreased the elasticity of the material and increased its strength—which is consistent with our findings.

This also increases the reliability of the results obtained in our study, as Qian and Li’s study emphasized the methods used to determine tensile strength and the importance of these methods in quality control [33].

One of the studies presented by Hashem and Mohamed showed that the interaction of MWCNTs with polystyrene can significantly influence the mechanical properties of the material. This study investigated the effects of nanoscale silica doping on mechanical properties and how these dopants influenced material performance [27]. Additionally, Li et al. [25] reported that the surfactant-assisted dispersion of MWCNTs significantly improves both the mechanical and thermal behavior of PS composites, which further supports the observed tensile improvements seen at 0.3 wt% in our study.

The hardness test of pure polystyrene and other reinforced composite materials was performed with the Sauter Shore Hardness Tester. The Shore method is used for hardness measurements of polymer matrix composite materials. In this method, the height reached by a hammer dropped in a vertical tube is measured when it hits the object and bounces back. The hardness of the material is directly proportional to this height measurement. The results of tensile and hardness tests of the composite samples obtained in this study are given in Table 1.

According to the hardness test results, the hardness of the composite materials decreased as the reinforcement ratio increased. The hardness of the composites remained constant at 98 Shore D for 0.1% and 0.2% MWCNT content and decreased only at 0.3 wt% reinforcement (95 Shore D). This suggests that, up to a certain threshold, the addition of nanotubes may not significantly disrupt the matrix structure [34]. However, at 0.3 wt%, more pronounced interfacial effects or agglomeration phenomena may emerge, reducing surface rigidity and slightly lowering the measured hardness. These results are consistent with those of Mathur et al. [21] and Li et al. [25], who observed similar trends in CNT-reinforced polymer composites. Consequently, enhancement of machinability appears to be more significant only at reinforcement levels beyond the threshold of 0.3 wt%, as observed in our study.

This study focused on multi-walled carbon nanotube (MWCNT) loadings up to 0.3 wt%. However, the results indicate a nonlinear response in mechanical behavior with respect to reinforcement content. While 0.3 wt% exhibited enhanced tensile and thermal properties compared to 0.1% and 0.2%, it is probable that, beyond a certain concentration, agglomeration of MWCNTs could occur, which would negatively impact mechanical integrity. Previous studies have shown that MWCNT loadings above 0.5–1.0 wt% in polystyrene matrices can result in dispersion issues, stress localization, and performance degradation [29,30]. Therefore, future studies should explore the optimal MWCNT concentration range in PS composites below this threshold through additional experimental or modeling efforts.

## 4. Conclusions

In this study, composite samples were prepared by adding different proportions of MWCNTs to polystyrene polymer. Their microstructural, physical, and thermal properties were characterized using four different techniques. Based on AFM analysis of composite samples, it was observed that RMS (Sq) and average roughness (Sa) values of pure PS, PS + 0.1% MWCNTs, PS + 0.2% MWCNTs, and PS + 0.3% MWCNTs were between 0.41 and 3.77 nm, while average roughness (Sa) values were between 0.33 and 3.25 nm. These AFM results show that the RMS value for MWCNT-reinforced composites decreased with increasing MWCNT weight percentage. The contribution of reinforcement materials to the composite surface was supported by these results. TGA results show that there was no change in the decomposition temperature of 0.1%- and 0.2%-doped MWCNT composites compared to pure polystyrene, starting at 298 °C and completed at 437 °C. However, the degradation of the 0.3% MWCNT composite increased up to 539 °C. These results showed that the thermal stability of 0.3% MWCNT composite was higher than pure PS and MWCNT reinforcement increased the thermal degradation temperature of the PS polymer. In addition to the microstructural characterization of the pure PS and PS/MWCNT composite materials, hardness and tensile tests were applied to these samples to determine specific mechanical properties. It is seen that both the elongation percentage and tensile strength values of the composite with 0.1% and 0.2% MWCNT reinforcement were 0.93% and 7.75 N/mm^2^, respectively. When the MWCNT ratio was increased to 0.3 wt%, the elongation percentage and tensile strength values of the composite were found to be 1.91% and 12.174 N/mm^2^, respectively. Tensile test results show that the elongation percentage and tensile strength values of the MWCNT-reinforced composites initially decreased compared to pure PS, but later reached a value close to pure PS for 0.3 wt% MWCNT. In conclusion, the thermal properties of pure PS were improved without damaging its mechanical properties by adding MWCNTs. With increased thermal properties, MWCNT-doped PS can be safely used in insulation materials and circuit boards, electronic device housings, and insulation parts.

In future studies, the effects of different MWCNT ratios and functionalization methods should be investigated in more detail. In addition, studies on the application areas of these composites should be carried out.

## Figures and Tables

**Table 1:** The results of average tensile and hardness tests with the standard deviations (all the tests were repeated five times).

Materials	The Percentage Elongation (%)	Tensile Strength (N/mm^2^)	Hardness (Shore d)
Pure PS	2.89 ± 0.07	14.15 ± 0.13	97.08 ± 0.27
PS + % 0.1 MWCNT	0.93 ± 0.03	7.75 ± 0.15	98.06 ± 0.20
PS + % 0.2 MWCNT	0.92 ± 0.03	7.75 ± 0.10	98.02 ± 0.24
PS + % 0.3 MWCNT	1.91 ± 0.05	12.17 ± 0.22	95.06 ± 0.34

## Data Availability

Data are contained within the article.

## Abbreviations

The following abbreviations are used in this manuscript:

PS	Polystyrene
MWCNT	Multi-walled Carbon Nanotube
AFM	Atomic Force Microscopy
TGA	Thermogravimetric Analysis
SEM	Scanning Electron Microscopy
FTIR	Fourier Transform Infrared
